# AGING MODULATION WITH EXERCISE AND MIMETICS

**DOI:** 10.1093/geroni/igad104.3173

**Published:** 2023-12-21

**Authors:** Dennis Lox

**Affiliations:** Florida Sports and Regenerative Medicine, Clearwater, Florida, United States

## Abstract

Understanding the molecular and cellular processes of the Longevity Pathway AMPK-SIRT1, and its activation by exercise and mimetics, plays a vital role in a series of molecular and cellular processes which stimulates optimal healthy cellular efficiency and functioning, ending with improved exercise tolerance. Knowledge of the role of exercise induced activation of the AMPK-SIRT1 pathway, and altering it towards dysfunctional processes and drivers of aging and disease propagation is important to understand to also understand. Cellular dysfunction unfortunately is the norm. This leads to molecular increase of P53 in a binding state not of DNA repair, but upregulation of gene transcription of NF-kB, which increases inflammatory cytokines, over activation of MTOR, and cellular senescence with secretion of the SASP. All these dysfunctions create feedback loops, which makes the process self propagating, resulting in progressive aging, and disease states. The most optimal state is regulation of the optimal molecular and cellular environment, via exercise or a mimetic stimulator of AMPK-SIRT1. Knowledge of the optimal body state for health, and enhancing this AMPK-SIRT process, is low body fat releasing beneficial adiponectin, and no inflammatory cytokines or adipokines. Training muscle releases positive myokines. Various mimetics can stimulate AMPK-SIRT1, such as Metformin, the mitochondrial derived peptide MOTS-C, AICAR, and other compounds intended for people with medical issues that prevents exercise. Some display a synergistic effect with exercise. Understanding, the benefits of exercise and modulation of aging, by encouraging healthy processes, and reducing the deleterious patterns is important to understand.

